# Retrovolution: HIV–Driven Evolution of Cellular Genes and Improvement of Anticancer Drug Activation

**DOI:** 10.1371/journal.pgen.1002904

**Published:** 2012-08-23

**Authors:** Paola Rossolillo, Flore Winter, Etienne Simon-Loriere, Sarah Gallois-Montbrun, Matteo Negroni

**Affiliations:** 1 Architecture et Reactivité de l'ARN, Université de Strasbourg, CNRS, IBMC, Strasbourg, France; 2 Institut Pasteur, Unité de Génétique Fonctionnelle de Maladies Infectieuses, Paris, France; 3 Inserm, U1016, Institut Cochin, Paris, France; 4 CNRS, UMR8104, Paris, France; 5 Université Paris Descartes, Sorbonne Paris Cité, Paris, France; University of Washington, United States of America

## Abstract

In evolution strategies aimed at isolating molecules with new functions, screening for the desired phenotype is generally performed *in vitro* or in bacteria. When the final goal of the strategy is the modification of the human cell, the mutants selected with these preliminary screenings may fail to confer the desired phenotype, due to the complex networks that regulate gene expression in higher eukaryotes. We developed a system where, by mimicking successive infection cycles with HIV-1 derived vectors containing the gene target of the evolution in their genome, libraries of gene mutants are generated in the human cell, where they can be directly screened. As a proof of concept we created a library of mutants of the human deoxycytidine kinase (dCK) gene, involved in the activation of nucleoside analogues used in cancer treatment, with the aim of isolating a variant sensitizing cancer cells to the chemotherapy compound Gemcitabine, to be used in gene therapy for anti-cancer approaches or as a poorly immunogenic negative selection marker for cell transplantation approaches. We describe the isolation of a dCK mutant, G12, inducing a 300-fold sensitization to Gemcitabine in cells originally resistant to the prodrug (Messa 10K), an effect 60 times stronger than the one induced by the wt enzyme. The phenotype is observed in different tumour cell lines irrespective of the insertion site of the transgene and is due to a change in specificity of the mutated kinase in favour of the nucleoside analogue. The mutations characterizing G12 are distant from the active site of the enzyme and are unpredictable on a rational basis, fully validating the pragmatic approach followed. Besides the potential interest of the G12 dCK variant for therapeutic purposes, the methodology developed is of interest for a large panel of applications in biotechnology and basic research.

## Author Summary

We exploited the error-prone replication machinery of HIV-1 and its ability to stably introduce transgenes in human cells to develop a novel system, Retrovolution, to generate libraries of mutants of cellular genes. When libraries are screened to isolate variants that modify the phenotype of the human cell for biomedical applications or basic research, false positives often arise from the classical screening procedures performed *in vitro* or in bacteria. Retrovolution allows an easy screening of the libraries directly in the human cell, where they are generated. We describe the creation and screening of a library of the *hdCK* (a human kinase activating several anticancer compounds) gene, to identify variants increasing the sensitivity of cancer cells to treatment with low, poorly toxic doses of the anticancer drug Gemcitabine. We isolated a dCK variant inducing death in tumour cells at doses up to 300 times lower than those required for killing non-engineered cells. The mutant presents mutations unpredictable on structural basis and revealed a change in enzymatic properties that accounts for the observed cellular effect. Besides the intrinsic interest of the mutant identified, these results fully validate Retrovolution as a mutagenesis system with broad applications in applied and basic research.

## Introduction

Broadening the repertoire of natural molecules and generating variants that confer new phenotypes to human cells are appealing perspectives for the development of biomedical applications, and for understanding fundamental cellular processes. To this end, in classical procedures, libraries of mutants are generated *in vitro* by degenerated PCR or DNA shuffling, and then screened on biochemical bases or with genetic tests in bacteria [1]. However, when the modification of human cells is sought, the mutants identified in these preliminary screenings often do not confer the desired phenotype due to differences in protein folding, post-translational modifications, and to the complex epistatic network that regulates the expression of the phenotype in the cells of higher eukaryotes. Alternatively, the library can be cloned in eukaryotic expression vectors for the screening step, albeit with the drawback of a considerable loss of complexity. The generation and screening of libraries of mutants directly in human cells would constitute an ideal solution to circumvent these problems. Nature provides organisms that are perfectly exploitable for this purpose: retroviruses. Indeed, after entry into the target cell, the viral polymerase (reverse transcriptase, RT) converts the viral genomic RNA, through an error-prone process that generates genetic diversity, into double-stranded DNA, which is then permanently integrated in the genome of the cell.

The human immunodeficiency virus (HIV-1) is the retrovirus with the strongest mutation rate, and constitutes therefore the ideal candidate for developing such approaches. During the process of Reverse Transcription, point mutations are introduced in the proviral DNA with a rate of 1–3.4×10^−5^ nt/cycle [2]–[4] and genetic diversity is further amplified by recombination [5], [6]. We report here the development of a new methodology (called “Retrovolution”) aimed at generating and screening libraries of cellular genes directly in human cells. In Retrovolution the error-prone replication machinery of HIV-1 is diverted to drive the evolution of cellular genes: by performing successive infection cycles of cell cultures with HIV-1-derived viral vectors containing the sequence target of the evolution inserted in their RNA, libraries of gene mutants are generated in human cells, where they can be directly screened for the desired phenotype. A system for the evolution of non-viral sequences based on the use of engineered HIV-1 had been previously developed for the optimization of the Tetracycline-regulated expression system [7]. In that system, the isolation of an improved phenotype was strictly linked to the efficiency of replication of the viral particle, whereas in Retrovolution selection is not linked to viral replication and the use of replication-defective, non-cytopathic vectors allows the isolation and expansion of cells with the desired phenotype, which is not possible when replication-competent HIV particles are used.

While relatively simple proteins or sequences, as a modified tag or reporter gene, have traditionally constituted the target for setting up evolution procedures [8], [9], to evaluate the power of the Retrovolution system we aimed at generating and isolating variants of a protein modifying a complex cellular phenotype, which could have a potential therapeutic interest. Namely, we wished to mutagenize the human deoxycytidine kinase (hdCK, NM_000788.2 mRNA NCBI accession number) gene with the goal of isolating an enzyme variant able to increase the sensitivity of cells to a widely used category of anticancer compounds, deoxycytidine (dC) analogues. The hdCK, physiologically involved in the phosphorylation of deoxycytidine, deoxyadenosine and deoxyguanosine in the salvage pathway for the deoxyribonucleotide biosynthesis, is a pivotal enzyme in the activation of deoxycytidine analogues used in chemotherapy (AraC and Gemcitabine, used in the treatment of leukemias and solid tumours). These compounds, administered as nucleosides, must be transformed by cellular enzymes in their triphosphate form to become active and induce apoptosis in the cell by interfering with DNA replication and repair [10]. The first phosphorylation step, catalyzed by the human deoxycytidine kinase, determines the overall efficiency of the process [11]. Accordingly, there is a direct correlation between the level of dCK expression and the sensitivity to deoxycytidine analogues in different types of tumor [12], [13]. The appearance of resistance forms among tumor cells, often due to a loss of dCK activity, and the toxicity of high concentrations of nucleoside analogues for non-tumor cells, constitute the major limits of the treatments with these compounds [14], [15]. The sensitization of cancer cells to low doses of drugs by insertion of a gene coding for a drug-metabolizing enzyme constitutes an appealing approach for gene therapy applications [16]–[18]. A dCK variant that could preferentially phosphorylate nucleoside analogues with respect to the natural substrate deoxycytidine, and therefore induce cell suicide upon exposure to low concentrations of prodrug, would constitute a good candidate for these “suicide gene” therapies in cancer treatment, but also in transplantation medicine. In this case, indeed, it would provide a human (and therefore poorly immunogenic) gene to use as negative selection marker to insert in transplanted cells to counteract their eventual uncontrolled proliferation *in vivo*.

So far, attempts at constructing a dCK mutant with improved ability in phosphorylating deoxycytidine analogues have been based on the information obtained from the 3D structure of the protein [19], [20]; these dCK variants based on the rational design displayed an overall increased catalytic activity, resulting in a highly improved affinity for the natural substrate and a less improved affinity for the prodrug, which is not predictive of the induction of a sensitization phenotype *in vivo*. By evolving the dCK gene with Retrovolution procedure we could directly screen for dCK variants inducing cell death in presence of doses of the deoxycytidine analogue Gemcitabine lower than the ones needed to kill cells bearing a wt dCK, and this allowed us to isolate a mutant consistently sensitizing cancer cells to the prodrug.

## Results

### The Retrovolution system

**Figure 1:**
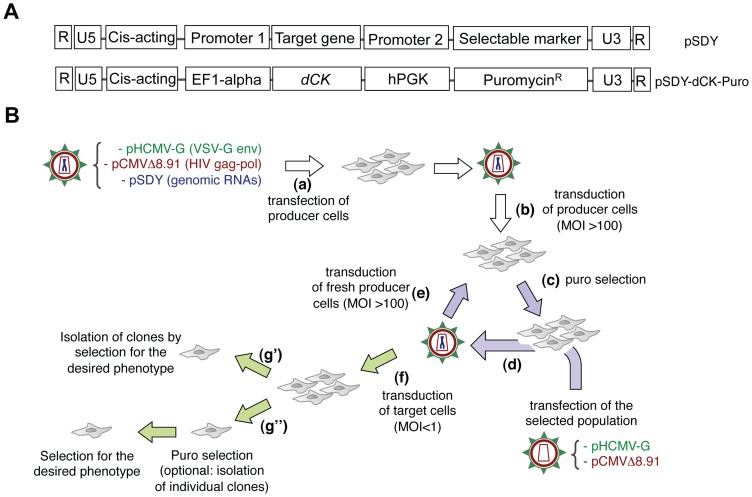
The Retrovolution system. *Panel A*. General structure of genomic RNAs for Retrovolution (top) and specific structure of the genomic RNA used in this study (bottom). Expression of the “target gene” is driven by an eukaryotic promoter (inducible or not, in this case the constitutive promoter of the human elongation factor 1-alpha, EF1-alpha); a selectable marker (here, the puromycin-resistance gene) is transcribed from a second, independent promoter (in this case the human phosphoglycerate kinase gene, hPGK) situated immediately downstream of the translation termination signal of the first gene. U3, R, U5 (LTRs) and HIV-1 cis-acting sequences are viral sequences necessary for viral genome packaging, reverse-transcription and integration. *Panel B*. Experimental procedure of Retrovolution. White arrows indicate the initial steps of the procedure, lilac arrows the steps involved in the generation of genetic diversity, and green arrows those involved in the selection steps. (a) HIV-based VSV-pseudotyped defective vectors are generated by transfection of producer cells (HEK-293T cells) with two plasmids leading to the production of the viral proteins (transcomplementation plasmids, pHCMV-G and pCMVΔR8.91), and one or potentially more plasmids leading to the synthesis of the genomic RNA (pSDY-dCK-Puro, outlined in panel A). The retroviral vectors generated (parental generation) are used to transduce (b) producer cells (HEK-293T) at a high MOI. Expression of the puromycin-resistance gene from proviral DNA allows the selection of the efficiently transduced cell population (c). Upon transfection of these cells with transcomplementation plasmids (d), new particles encapsidating the gRNA transcribed from the proviral DNA inserted in the cell's genome will be generated and used to transduce fresh producer cells (e). Steps (c), (d), and (e) are reiterated, producing subsequent viral generations (each cycle requires a week in the case of puromycin selection, but this time varies with the selectable marker used). To screen the library, some of the vectors are used to transduce the appropriate target cells at a low MOI (f). Transduced cells can then be directly screened on the basis of the desired phenotype (g′) or they can first be selected for the expression of the selectable marker (with the possibility of isolating individual clones or not) and subsequently selected based on the expression of the desired phenotype (g″).

**Figure 2:**
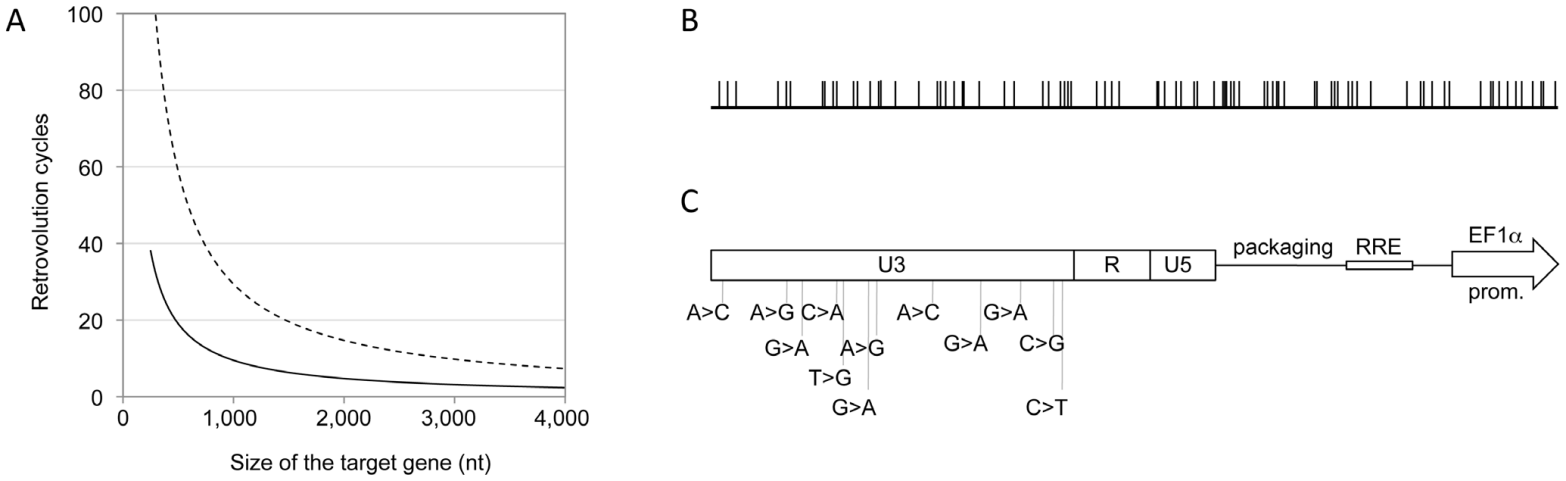
The system generates genetic diversity. *Panel A*. Expected relationship between the size of the target gene and the number of Retrovolution cycles required to obtain a library containing one mutated position per virion in the gene sequence, based on the mutation rate of 3.4×10^−5^ mutations per nt per cycle, described in the literature [3] (dashed line), and based on the rate observed experimentally in this work (solid black line). *Panel B*. Distribution of the mutated positions within the dCK coding sequence (783 bp) found by sequencing 40 clones at generation 16. *Panel C*. Position of the 12 mutations detected in the U3 region of the LTRs of all vectors sequenced starting from generation 8 (included).

The Retrovolution system, outlined in Figure 1, is based on the insertion of the gene target of the evolution process in the genomic RNA (gRNA) of conditional replication-defective, HIV-1 derived vectors pseudotyped with the Vesicular Stomatitis Virus envelope (VSV-G) (Figure 1A). Repeated cycles of transduction of producer cells with these vectors, as indicated in Figure 1B, mimic successive infection cycles by HIV-1, during which mutations will be inserted in the gRNA and re-shuffled by recombination [21], [22], an event favored when transductions are performed at a high Multiplicity Of Infection (MOI). During the procedure, HIV-1 Reverse Transcriptase will introduce mutations all along the viral gRNA and, while those falling in functional regions of the vector will be selected (positively or negatively), the others, including those falling in the target gene, will accumulate in an unbiased fashion. This process generates a library of mutants of the target gene directly inserted into the vector tasked with delivering the transgene to the human cell, and requires minimal intervention from the experimenter. During the evolution procedure, cells that did not receive the viral vector and therefore do not contain an integrated proviral DNA are removed by puromycin treatment (the puromycin resistance gene being inserted as a selectable marker in the gRNA of the vectors, see Figure 1A). As an alternative, GFP can be used as a selectable marker instead of *puro^R^* and successfully transduced cells can be sorted by FACS at every round: the use of GFP would fasten the evolution procedure (a complete puromycin selection takes three to four days), but would require a much more important manual intervention. The average number of positions that will be mutated in the target gene will depend on the number of “infection cycles” performed, on the size of the target gene itself, and on the mutation rate of the reverse transcription process, as outlined in Figure 2A. Once reached the ideal complexity, the library of mutants will be screened by transducing target cells (the human cells to which the intended phenotype is to be conferred) at a low MOI (≪1) to minimize the possibility that one cell will receive more than one vector, followed by clonal analysis (Figure 1B). As the vectors are pseudotyped with the VSV envelope, which can infect virtually any kind of mammalian cells, the library can be screened in all human cell types.

### Retrovolution generates genetic diversity

A library of dCK variants was generated by using the dCK coding sequence (dCK-cs) as the target gene (Figure 1A) of the Retrovolution procedure. By sequencing part of the library after 9 cycles of transduction we could calculate an ongoing mutation rate of 1×10^−4^ nt/infectious cycle. Based on this, the library was screened upon reaching generation 16, at which a complexity of 1.5 mutated positions per dCK-cs (783 bp) was expected (Figure 2A). A further sequencing of a subset of the library at this generation confirmed the presence of 1.4 mutated positions per dCK gene (Table 1). In the dCK variants sequenced all kinds of mutations were present with a bias toward G>A transitions (62.3%), consistent with the mutational pattern of HIV-1 RT [23]. While the sequencing of the dCK gene at different generations of the Retrovolution system confirmed that mutations were inserted randomly along the gene (Figure 2B), the sequencing of the viral backbone of the corresponding clones revealed a consensus of 12 mutations in the U3 region of each vector (Figure 2C). These consensus mutations appeared in the promoter region of HIV-1 LTR since the F8 generation and were fixed in the population ever since. Moreover, their nature diverged from the typical HIV-1 mutation pattern since the percentage of G>A transitions was consistently lower (33%). No mutations were instead found in the other cis-acting regions essential for genome packaging, reverse transcription, integration and nuclear export. Overall, this strongly suggests that the viral sequences underwent selection for an optimized transcription of the genomic RNA from the U3 promoter in the specific context of the producer cells, while preserving the functionality of the vector, and allowing the production of a library of randomly mutated variants of the target gene.

**Table 1:** Mutation frequencies for the dCK gene at different steps of the Retrovolution procedure.

Generation	N° of sequenced clones	N° mutated positions	Relative mutation rate	N° of mutations per clone	Average mut positions/clone
F8	16	14	1.2×10^−4^	0–5	0.88
F16	40	61	1.14×10^−4^	1–9	1.4

### Identification of mutants of the human dCK conferring an increased sensitivity to Gemcitabine

**Figure 3:**
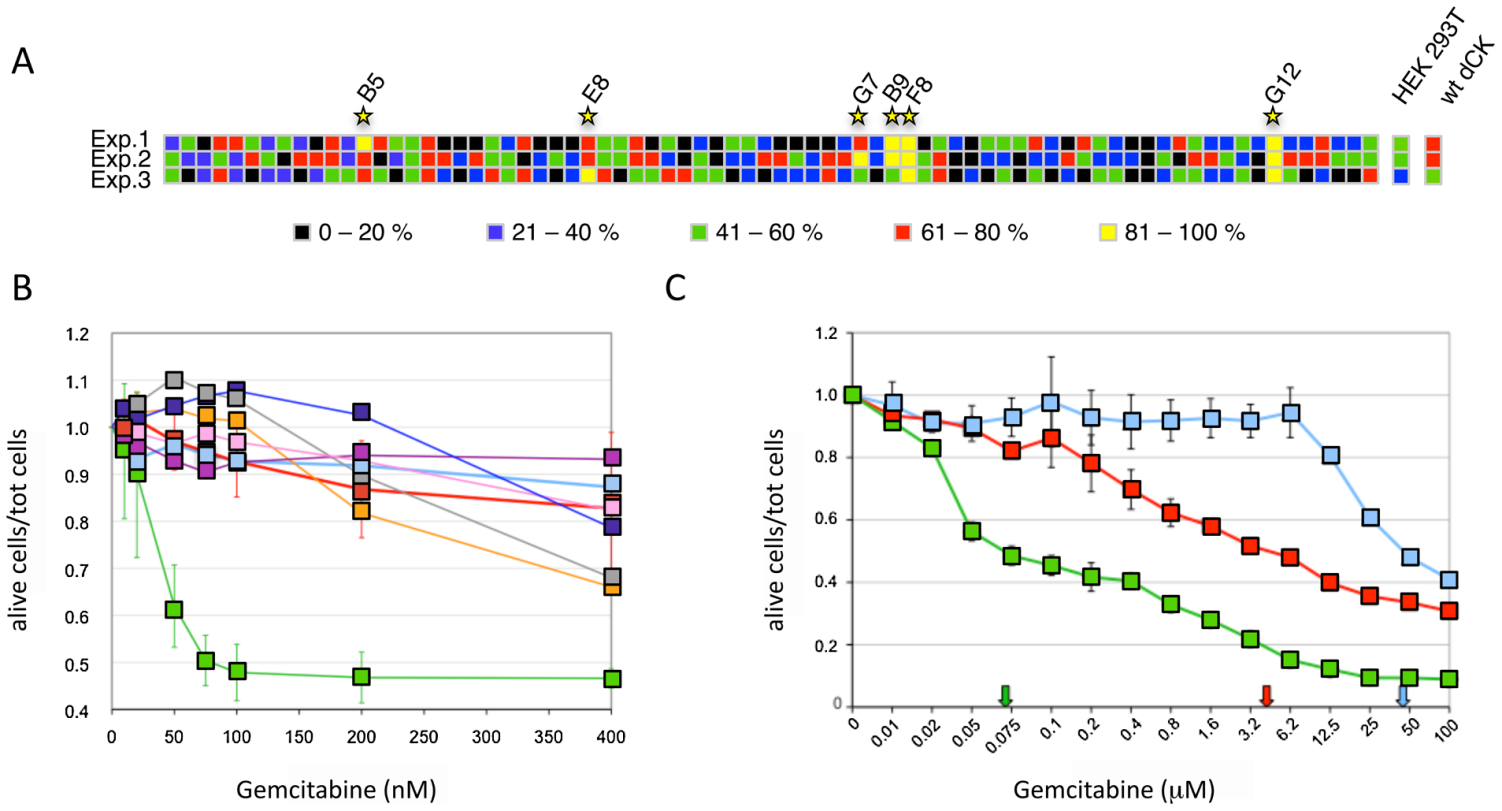
Screening of the dCK library. *Panel A*. Viability of 76 isolated clones of HEK-293T transduced with the dCK-F16 viral library at a MOI<1 in the presence of 10 nM Gemcitabine. The different colors represent the percentage of dead cells on the total of seeded cells. Each line corresponds to an independent experiment, and each column to one clone. The results obtained with untransduced cells and cells transduced with the wt-dCK gene are given on the right (“HEK-293T” and “wt-dCK”, columns, respectively). *Panel B*. Viability of selected Messa10K “polyclonal populations” in the presence of increasing concentrations of Gemcitabine. Thick pale blue line, untransduced Messa10K cells; thick red line, cells transduced with wt-dCK; thin lines, cells transduced with the vectors derived from the clones identified in HEK-293T (Figure 1D): pink, B9; gray, B5; dark blue, F8; purple, G7; orange, E8; green, G12. Values represent the ratio of the number of live cells over the total of cells seeded for each Gemcitabine concentration. For each dCK variant, 4 to 9 populations of Messa10K cells were generated independently, as described in Figure S1, and for each population the experiment was carried out in duplicate. For the sake of clarity, the error bars are shown only for samples G12 and wt-dCK. The values of G12 and wt-dCK are significantly different for every Gemcitabine concentration tested (p<0.0001). *Panel C*. Cell death for Messa10K “polyclonal populations” either non-transduced (light blue) or transduced with the wt-dCK (red) or the G12 variant (green), with a larger range of Gemcitabine concentrations than in panel B. The arrows indicate the IC50 values for Gemcitabine in the three different cell types.

To screen the library for the presence of dCK variants that confer an increased sensitivity to low concentrations of the deoxycytidine analogue Gemcitabine, viral vectors from generation 16 were used to transduce HEK-293T cells at a low MOI (MOI = 0.03) to ensure that each cell did not contain more than one variant of the transgene, and single clones were isolated by limiting dilution and puromycin selection. We then measured the viability of 76 individual clones in the presence of increasing concentrations of Gemcitabine (10, 35 and 70 nM) and calculated the cell death rate as the number of alive cells at concentration X of Gemcitabine divided by the number of cells at 0 Gemcitabine. The experiment was repeated three times using, as controls, HEK-293T cells either untransduced or transduced with a wt-dCK containing vector. The two higher Gemcitabine concentrations tested resulted to be too strong for an effective screening since all samples, including controls, displayed more than 80% of cell death (data not shown). At 10 nM Gemcitabine, instead, 6 of the 76 clones tested yielded, in at least one of the three independent experiments, a cell death rate higher than both untransduced HEK-293T cells and cells bearing the wt dCK transgene (Figure 3A). These six clones were selected for further analysis.

### The mutant G12 sensitizes different cancer cell lines to Gemcitabine

The 6 clones isolated in the preliminary screening were further characterized for the ability of their transgenes to induce sensitization to Gemcitabine in the Messa10K cell line, uterine sarcoma cells that express an inactive dCK and are, therefore, highly resistant to this drug [13]. These cells provide an ideal background for testing the ability of the library of dCK mutants to increase sensitivity to Gemcitabine, since the phenotype generated by the introduction of a single copy of a dCK variant will not be masked by the activity of the wt dCK protein present in the cells. For each HEK-293T clone selected, viral particles were rescued by transfection with the plasmids encoding HIV-1 Gag-Pol and VSV Env, and used to transduce Messa10K cells at a MOI<1. Since the effect induced by a transgene on the cell phenotype can be strongly influenced by the position of integration of the proviral DNA within the target cell genome, transduction of the Messa10K cells was performed following a procedure, outlined in Figure S1, that generates, for each transgene, a population of cells containing the same transgenic sequence inserted at different genomic locations (“polyclonal population”). For each dCK variant analysed we established 4 to 9 independent Messa 10K populations with this procedure, that allows to average the possible effects of the insertion site and to highlight the phenotype induced by the transgenic sequence itself. From the analysis of cell death ratios of Messa 10K populations in presence of increasing concentrations of Gemcitabine appeared that one of the 6 transgenes identified on HEK-293T cells, G12 (E171K, E247K, L249M), strongly sensitized cells to the drug. In the 9 G12-Messa10K polyclonal populations tested, indeed, 50% of the cells died at 75 nM Gemcitabine, while at the same concentration only 10% of Messa 10K containing a wt dCK were killed (Figure 3B). The analysis of a broader range of Gemcitabine concentrations revealed that in G12-Messa 10K cells the Gemcitabine IC50 was reduced by 60-fold compared to cells transduced with wt-dCK, and by 300-fold relative to untreated cells [13] (Figure 3C).

**Figure 4:**
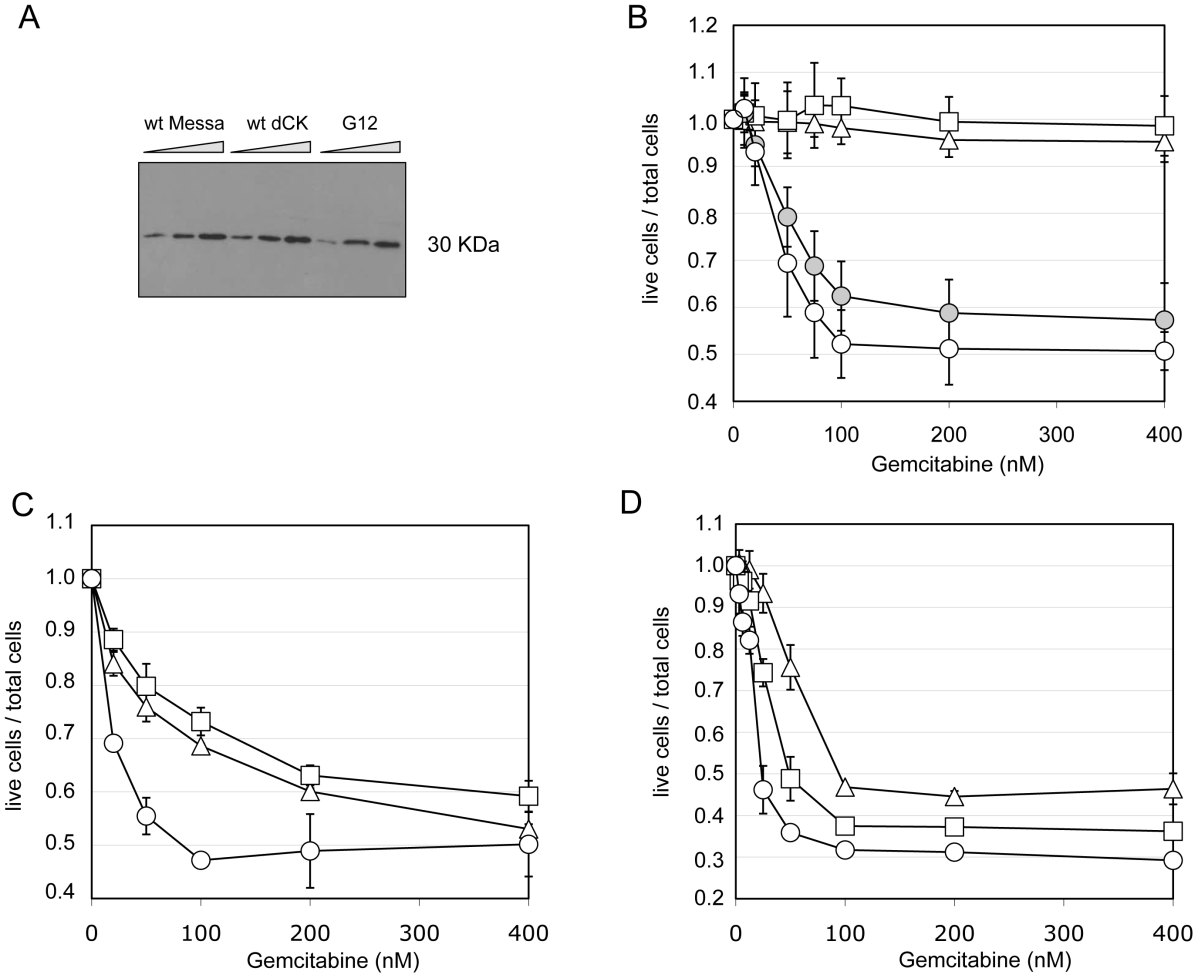
The sensitization phenotype is due to a modified dCK activity. *Panel A*. Western Blot analysis of dCK expression levels in different “polyclonal populations”. M: untransduced Messa10K cells; M-dCK: 4 Messa10K populations transduced with wt-dCK gene; M-G12: 4 Messa10K populations transduced with the G12 variant. For each population 6,12 and 24 µg of total protein were loaded on the gel. *Panel B*. Gemcitabine sensitivity of Messa10K cells transduced with vectors bearing the G12 mutant sequence inserted in a wild-type backbone. Triangles: untransduced cells. Squares: cells transduced with wt-dCK. Empty circles: cells transduced with G12. Full circles: cells transduced with G12/wt-backbone. Results are the average of 6 independent experiments. *Panels C and D*. Sensitivity to Gemcitabine of HT29 cells (panel C) and of BxPC3 cells (panel D) transduced with one copy of G12 variant per cell. Triangles: untransduced cells. Squares: cells transduced with a wt-dCK vector. Circles: cells transduced with a G12 vector. Results are the average of 2 independent experiments.

As shown by Western Blot (Figure 4A), sensitization of Messa10K cells by G12 is not the consequence, as could result from mutations in the EF1-alpha promoter (Figure 1A), of a higher level of protein expression compared to wt-dCK. Nevertheless, sequencing of the entire G12 gRNA revealed the presence of mutations within as well as outside the dCK-cs and these mutations could influence the phenotype observed. To rule out this possibility we inserted the G12 dCK-cs in a wild-type gRNA backbone plasmid (generating “G12/wt-backbone” gRNA), and tested the deriving viral vectors on Messa10K cells. Also in this case a sensitization to the nucleoside analogue (Figure 4B) was detected, indicating that the Gemcitabine sensitization phenotype is rather due to properties of the dCK variant encoded by G12 than to mutations arisen in other portions of the genomic RNA.

Messa10K cells constitute a model of Gemcitabine-resistant cells lacking an active endogenous dCK, as frequently emerge during chemotherapy [15]. To investigate the effect of the insertion of G12 in tumour cells that express an endogenous functional dCK protein, we assessed the phenotype conferred by the insertion of the mutated dCK to the colon carcinoma cell line HT29, and to the pancreatic cancer cell line BxPC3. In HT29, sensitivity to Gemcitabine is strictly linked to the levels of dCK activity, whereas in BxPC3 the sensitivity to the prodrug depends on a different mechanism, the expression levels of the cytidine deaminase CDA [24], [25], an enzyme that inactivates the fully phosphorylated Gemcitabine by deamination. Therefore, while in the former cells the insertion of an improved variant of dCK should lead to a significant sensitization to the drug, in the latter ones the effect should be reduced. We generated two independent G12 polyclonal populations for each cell line and tested them. Consistently with our hypothesis, we observed a considerably stronger effect of G12 in HT29 than in BxPC3 (Figure 4C and 4D, respectively), with a decrease in the Gemcitabine IC50 with respect to cells containing a wt-dCK from 1.6 µM to 80 nM in HT29 and only of less than two folds (from 40 to 22 nM) in BxPC3. These results support the idea that the Gemcitabine sensitization phenotype is due to an improved ability of the G12 mutated kinase to activate the prodrug in cell culture and underscores that the insertion of a single copy of the transgene can increase the sensitivity of cells to Gemcitabine even in the presence of a wt dCK activity.

### Change in substrate specificity of the G12 mutant

**Figure 5:**
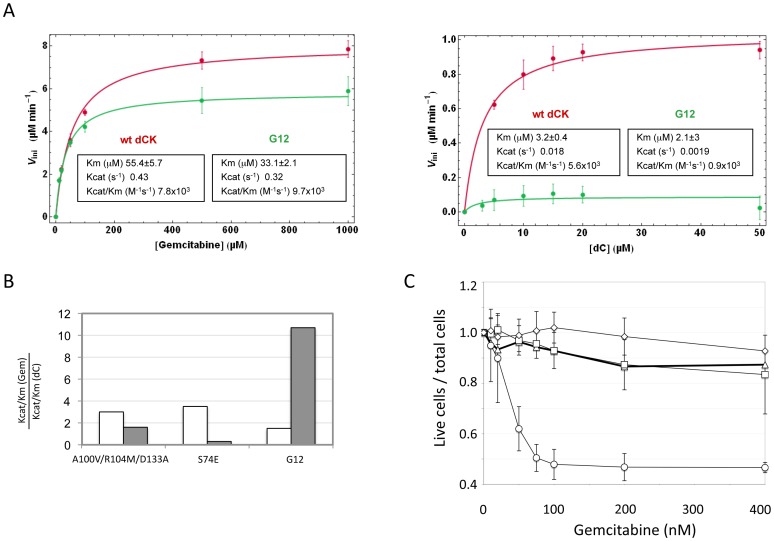
Biochemical characterization of G12. *Panel A*. Phosphorylation kinetics of wt-dCK and G12 mutant overexpressed in *E.coli*. Steady state kinetic data of wt and G12 dCK fitted according to the Michaelis-Menten equation. *Panel B*. Ratio of the values of K_cat_/K_m_ for gemcitabine (Gem) with respect to the values of K_cat_/K_m_ for the natural substrate dC for the triple mutant A100V/R104M/D133A, described in reference [19], for the S74E mutant, described in reference [20], and for the G12 mutant, described here. The value found in each of the three studies for the wt dCK is given in white, while that of the respective mutant is given in grey. *Panel C*. Viability of selected Messa10K “polyclonal populations” transduced with vectors containing the wt dCK (squares), mutant G12 (circles), or the triple mutant A100V/R104M/D133A [19] (diamonds), and of untransduced Messa 10K cells (triangles), in the presence of increasing concentrations of Gemcitabine.

To further support the existence of a link between the effect of the mutant in cell culture and its enzymatic activity, we expressed in *E. coli* the G12 mutant and the wt type dCK proteins, purified them and characterized their efficiency of phosphorylation of Gemcitabine and of dC *in vitro*. While Gemcitabine was phosphorylated with comparable efficiencies by the wt and G12 dCKs (left panel of Figure 5A), the natural substrate dC was phosphorylated at almost undetectable levels by G12, with a dramatic drop with respect to the efficiency of phosphorylation observed with the wt dCK enzyme (right panel of Figure 5A). The G12 dCK mutant therefore displays an altered substrate specificity, constituting a kinase that specifically phosphorylates Gemcitabine, even in the presence of the natural substrate of wt dCK. These observations underscore that, *in vivo*, an increased sensitivity to the prodrug can be efficiently achieved through a decreased ability of the kinase to phosphorylate its natural substrate, rather than through an improved efficiency of phosphorylation of the prodrug itself. So far, efforts at improving the efficiency of phosphorylation of nucleoside analogues by dCK have relied on the rational design of mutants either of the active site region [19] or of Ser74, the phosphorylation of which has been described to modulate the dCK enzymatic activity *in vivo* [20], [26]. Although the resulting enzymes displayed an increased overall catalytic activity *in vitro*, the relative efficiency of phosphorylation (expressed as the ratio K_cat_/K_m_Gemcitabine/K_cat_/K_m_dC) of the drug with respect to the natural substrate was decreased compared to the wt kinase, a trend opposed to that observed for the G12 mutant we describe (Figure 5B). Consistently with the view that these mutants should not impact sensitivity of cells to Gemcitabine treatment, when we constructed a vector carrying the sequence of the triple mutant A100V/R104A/D133A [19] and used it to transduce Messa10K cells, no altered sensitivity to Gemcitabine was observed (Figure 5C).

## Discussion

We describe here the development of a new system of mutagenesis of cellular genes aimed at identifying genetic variants of interest for applications in basic and applied research. A crucial feature of the system is the possibility of performing, easily and in a controlled manner, a straightforward screening of the library in the human cell. This pragmatic approach allows to overcome the generation of false positives variants, a frequent problem encountered upon *in vitro* screening of libraries. The application of the Retrovolution system led to the identification of a dCK mutant that fulfils the long-sought feature of increasing sensitivity of tumour cells to Gemcitabine treatment, based on the presence of mutations unpredictable on a rational basis. The screening step was performed following a protocol that ensures that the isolated mutant sensitizes the cells independently from the integration site of the provirus, therefore constituting a good candidate as “suicide gene” in gene therapy.

The mutagenic potential of Retrovolution relies on the error-prone nature of the replication machinery of HIV-1, and on the frequent occurrence of recombination, that reshuffles the pre-existing mutations contributing to increase the diversity of the library. We show here that these two sources of genetic diversification prompt enough diversity as to lead to the identification of mutants of interest. With a relatively reduced number of cycles the system allowed the creation of a library in which each variant of the target gene was characterized by 1.5 mutated positions on average. A higher proportion of mutations would have increased the chances of producing a high percentage of non-functional proteins. An alternative approach for generating a library of mutants of cellular genes contained in lentiviral vectors would have been constituted first by a mutagenesis step of the cellular gene through conventional methods as error-prone PCR, followed by the insertion of the library in the lentiviral gRNA plasmid. With respect to this approach, the method we describe, while slower in the generation of a complex library, provides the advantage of circumventing the unavoidable loss of complexity inherent to the step of cloning the library in the lentiviral gRNA plasmid. Another way to accelerate the acquisition of mutations would have been the use of error-prone reverse transcriptases or mild mutagens. These procedures were not privileged, though, due to the inherent drawback of reduced yield of vector production [27], [28].

Depending on the nature of the target gene, the experimental settings of Retrovolution can be adapted to introduce selection ongoing during the mutagenesis steps. In the present work this was not possible since we targeted a gene for which a negative selection, as the induction of cell death, needed to be applied. The targeting of the dCK gene was aimed, despite the intrinsic difficulty of the screening procedure, at the isolation of variants of a gene with a relevant biological interest for biomedical applications. The properties of the mutant identified, indeed, constitute the second major point emerging from this work.

Improving the efficiency of the currently clinically employed anticancer drugs and overcoming resistances arising during treatments is a major goal of cancer research. To improve the efficiency of the treatments with nucleoside analogues like Gemcitabine, gene therapy approaches aimed at sensitizing cancer cells through the introduction of a transgene that improves the efficacy of drug activation have been proposed. The hdCK is the best candidate transgene for this purpose as the kinase is responsible for the limiting step in intracellular activation of deoxycytidine analogues currently used in clinical treatments. The competition between the natural substrate of dCK and the prodrug inside the cell constitutes an obstacle to the efficient activation of the prodrug itself. The rational design of mutants in the catalytic site of the kinase attained the goal of improving phosphorylation of the prodrug in the past, but the mutants isolated displayed a concomitant, and stronger, increase in the efficiency of phosphorylation of the natural substrate [19], [20]. As a result, phosphorylation of the prodrug is expected to remain disfavoured with respect to the natural substrate and these mutants fail, as we confirmed (Figure 5C), to confer a drug sensitization phenotype to the cell. The G12 mutant, on the contrary, has an increased specificity for the phosphorylation of Gemcitabine due to the fact that it completely lost the ability to phosphorylate its natural substrate. This results in a reduction of the competition between the two substrates *in vivo* and in a sensitization of the cells to the prodrug. The biochemical mechanism through which an increased sensitization to the drug is obtained is therefore opposed to what generally tried to obtain by engineering the dCK on a rational basis.

The screening for the isolation of the G12 mutant was performed on Messa 10K cells, where the most striking sensitization was observed. These cells constitute a model of tumoral cells acquiring resistance to deoxycytidine analogues due to a loss of dCK activity, a fundamental problem that has to be bypassed for an improvement of cancer treatment. Nevertheless, the G12 mutant has an effect also on tumour cells presenting an endogenous dCK activity, like HT29 and HEK-293T. Therefore, besides a potential application in cancer treatment, G12 also constitutes a promising suicide gene to use as negative selection marker in cells used in transplantation medicine. Suicide genes are inserted in cells transplanted for therapeutical purposes to specifically ablate them in the event they would undergo uncontrolled proliferation *in vivo*. To this end exogenous genes are generally used, the most frequently employed being the Herpes Simplex Virus thymidine kinase gene in association with gancyclovir treatment. A problem of increasing relevance in clinical gene therapy, though, is constituted by the immune response raised by the patient against the protein encoded by the suicide gene [29], [30]. A suicide gene of human origin, as the mutant described here, is expected to have a faint possibility of being highly immunogenic and therefore represents an ideal candidate for this application.

**Figure 6:**
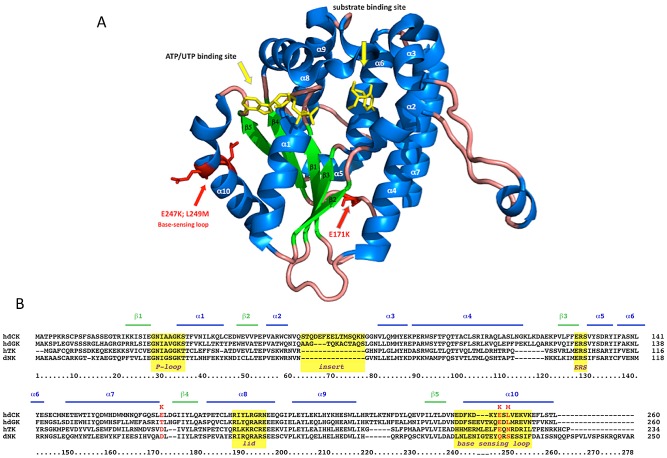
G12 aminoacidic substitutions. *Panel A*. Diagram of a dCK monomer [38] designed with PyMol on 2N01 file of PDB. alpha-helices are in blue, beta-sheets in green, phosphate donor and substrate in yellow. G12 mutations are highlighted in red and indicated by red arrows. *Panel B*. ClustalW sequence alignment of human dCK, dGK, TK2 and of *Drosophila melanogaster* dNK. Yellow boxes highlight well-known structural elements of the protein.

Besides conferring an increased sensitivity to Gemcitabine through an unexpected mechanism, the G12 mutant is characterized by mutations localized in regions unpredictable on a rational basis. The human dCK is a globular, dimeric protein in which each monomer is formed by a core of 5 beta-sheets surrounded by 10 alpha-helices [19] (Figure 6A). G12 dCK carries the mutations E171K, E247K, and L249M, the first two of which induce a charge change in conserved residues (Figure 6B). Amino acids 247 and 249 are located in the “base-sensing loop” (alpha-helix 10), which influences the folding of the protein upon binding of ATP or UTP as phosphate donor [26], [31] and, consequently, also affects substrate binding. Indeed, a variant containing only these mutations, which was identified during the screening of the library (mutant E8, Figure 3B), slightly increased Messa10K cells sensitivity to Gemcitabine treatment, although with a cell death ratio that did not significantly differ from that of the wt-dCK (p>0.05). The marked sensitization observed with G12 thus requires the presence of the additional mutation, E171K. Residue 171 is located at the base of alpha-helix 7 that, with alpha-helix 4, is involved in the generation of the interface of the dCK dimer [19], and its mutation in a residue of opposite charge could potentially influence the efficiency of formation of the active dimer itself. Gel filtration analyses, though, revealed that the extent of dimerization of G12 does not differ from that of the wt-dCK (Figure S2). It is therefore likely that, as observed for other proteins [32], [33], the mutation rather triggers long-distance changes in the quaternary arrangement of the protein, possibly involving the region of the active site.

In conclusion, Retrovolution allowed the identification of a promising suicide gene to use in cancer treatment but also as negative selection marker in transplantation medicine by allowing isolating a variant of the dCK that would not have been predictable on a rational basis. The property of producing the library and allowing its screening directly in human cells, ensuring that each mutant emerging from the procedure will be relevant for modifying the phenotype of the cells, is central to these findings. Retrovolution thus opens new avenues for the modification of genes conferring complex phenotypes of interest for a broad field of applications in basic and applied research. With the exploitation of its combination of genetic flexibility and ability to deliver transgenes to human cells, the lifestyle of one of the most important pathogens of our recent history appears far from being fully exploited.

## Materials and Methods

### Cell lines

HEK-293T cells were obtained from the American Type Culture Collection (ATCC) and grown in Dulbecco's Modified Eagle's Medium (Gibco) supplemented with 10% FBS and 100 U/ml pennicillin-100 mg/ml streptomycin. Messa10K cells were kindly provided by LP. Jordheim and grown in RPMI medium supplemented with 10% FBS and penicillin-streptomycin. HT29 cells were kindly given by JN. Freund and grown in DMEM+ 10% FBS. BxPC3 cells were kindly provided by M. Dufresne and grown in RPMI+ 10% FBS. All cells were incubated at 37°C+5% CO_2_.

### Viral vectors production and transduction

For the creation of the first generation of viral vectors, 8 plates containing 5×10^6^ HEK-293T cells were transfected using the calcium phosphate protocol with 10 µg of pCMVΔR8.91 plasmid [34], 5 µg of pHCMV-G plasmid [34]–[36] and 10 µg of genomic plasmid sdy-dCK (encoding the RNA outlined in Figure 1A). 48 h after transfection, the virus-containing supernatant was collected, filtered through a 0.45 µm filter and concentrated 40-fold in Vivaspin 20 columns (MWCO 50 KDa, Sartorius Stedim Biotech). Transfections for the creation of subsequent generations were performed with 10 µg of pCMVΔR8.91 and 5 µg of pHCMV-G plasmid. Transductions during the evolution procedure were performed on 5 million HEK-293T cells with 1 ml of 40× viral vectors (MOI>100). Transductions for the isolation of single clones were performed with 1 ml of 1∶500 dilution of viral vectors (MOI<1) on 3.5×10^6^ HEK-293T cells; after transduction, cells were seeded at 100 cells/well in 96-well plates in DMEM+ puromycin. For the generation of polyclonal populations of target cells, transduction was performed on 1×10^6^ cells with 1 ml of 1∶5 diluted viral vectors rescued from the isolated clones. Transduced cells were selected by adding puromycin 24 h after transduction using 0.6 µg/ml to HEK-293T cells, 0.5 µg/ml to Messa10K cells, 0.8 µg/ml to HT29 cells and 0.4 µg/ml to BxPC3.

### Amplification and sequencing of the dCK transgene

Clones of the F8 and F16 generation of the dCK library that had been isolated by limiting dilution were expanded in 10 cm plates and pelleted. Cells were lysed with 250 µl of Cell Direct PCR (VIAGEN). The dCK transgene was amplified from 1 µl of the lysate with oligonucleotides on the vector, EF1 (5′-gatgtcgtgtactggctccg-3′) and PGK (5-gatgtggaatgtgtgcgagg-3′), flanking the transgene. Sequencing of the PCR fragment was performed by the GATC sequencing service.

### Library complexity calculations

The curves for the calculation of the number of Retrovolution cycles needed to have one mutation per target gene (Figure 2A) were drawn based on the formula *p*/*m×n* where *p* = n°of mutations wanted per target gene, *m* = mutation rate/nt, *n* = size of the target gene in nt.

### Gemcitabine sensitivity test

Cells were seeded at 5000 cells per well in a 96-well plate and grown overnight at 37°C. Two rows were used for each population. 12 h after seeding, increasing concentrations of Gemcitabine (0–400 nM or 0–100 µM) were added to each well and incubation was continued for an additional 72 h. Cell viability was measured by MTT test (CellTiter 96 Non-Radioactive Cell Proliferation Assay, Promega) and the number of living cells in each well was evaluated by measuring the OD at 570 nm. For each population, the fraction of living cells was calculated as OD570 concentration X Gem/OD570 concentration 0 Gem, to estimate the sensitivity of the population to the prodrug.

### Western blot analysis of dCK production in different populations

Messa10K cells transduced with the different dCK variants were lysed in 1X RIPA buffer, and 6, 12, 24 µg of total protein (evaluated by Bradford) for each cell type were loaded on a 12% bis-tricine gel (Invitrogen). After transfer on a PVDF membrane, the dCK proteins were analysed by western Blot with 1∶4000 dilution of a polyclonal anti-dCK antibody (rabbit, Sigma-Aldrich) and 1∶3000 anti-rabbit HRP (BioRad) conjugated secondary antibody and detected by autoradiography.

### Statistical analysis

To evaluate whether the average amounts of cell death occurring in the Messa10K populations containing different dCK variants were significantly different from the value shown by Messa10K wt-dCK, a two sample t-test was applied for the different concentrations of Gemcitabine.

### Protein overexpression and purification

The wt-dCK and G12 sequences were cloned in the pET14b plasmid and expressed in *E. coli* cells BL21 DE3 pLysE. Protein expression was induced by adding 0.1 mM IPTG, and cells were collected after 4 h of growth at 37°C. His-tagged proteins were eluted with 250 mM Imidazole from His-Trap TM FF Columns (GE HealthCare), the Histidine tag was removed using the S-Tag Thrombin Purification Kit (Novagen), and dCK and G12 were further purified by gel filtration on S-200 Sephacryl columns (GE Healthcare). Purified proteins were used for the dCK activity assay or stored at −80°C in 30% glycerol.

### dCK phosphorylation activity

The efficiency of phosphorylation of the natural substrate deoxycytidine and of the prodrug Gemcitabine were measured for purified wt-dCK and G12 in a NADH-based assay as previously described [37]. All reagents were purchased from Sigma (France) except Gemcitabine (Lilly France SAS). Enzymes were assayed at RT at a concentration of 0.3 µM with Gemcitabine or 0.9 µM with dC. Gemcitabine was used at concentrations between 10 µM and 1 mM and dC at concentrations between 5 and 50 µM. ATP was 4 mM. All experiments were performed in triplicate.

## Supporting Information

Figure S1 Screening procedure used for limiting the influence of the retroviral insertion site and of insertional mutagenesis on the expression of the cell phenotype. The first characterization of the individual clones of HEK-293T cells, reported in Figure 3A, was carried out on isolated clones whose cells contained a single transgene inserted in the same genomic location. Each clone was transfected with the plasmids encoding the viral proteins, to generate a population of vectors that carried the same dCK variant. These vectors were used to transduce cells (Messa10K cells) at an MOI≪1, giving rise to 20–100 clones for each transgene, which were mixed and expanded, resulting in a population of cells bearing the same dCK variant inserted in different genomic sites (“polyclonal population”). For each HEK-293T clone selected, 4 to 9 independent “polyclonal populations” of Messa10K cells were created and analyzed.
(PDF)

Figure S2 Gel filtration elution profile of wt and G12 dCK proteins.
(PDF)
