# Supplementary figures and images for: Retrovolution: HIV–Driven Evolution of Cellular Genes and Improvement of Anticancer Drug Activation
Source: PLoS Genet. 2012 Aug 23;8(8):e1002904. doi: 10.1371/journal.pgen.1002904 (PMC3426553; doi:10.1371/journal.pgen.1002904)

**Fig. S1**

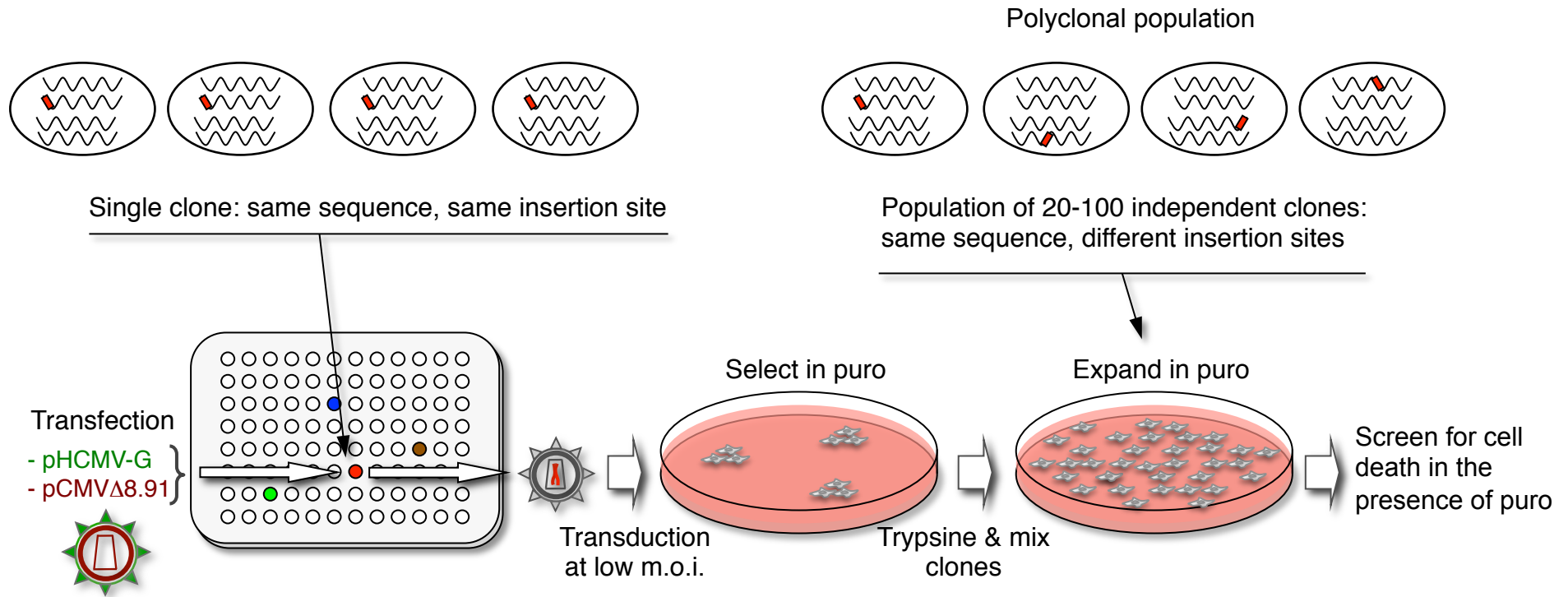

Supplement: Figure S1 — Screening procedure used for limiting the influence of the retroviral insertion site and of insertional mutagenesis on the expression of the cell phenotype. The first characterization of the individual clones of HEK-293T cells, reported in Figure 3A, was carried out on isolated clones whose cells contained a single transgene inserted in the same genomic location. Each clone was transfected with the plasmids encoding the viral proteins, to generate a population of vectors that carried the same dCK variant. These vectors were used to transduce cells (Messa10K cells) at an MOI≪1, giving rise to 20–100 clones for each transgene, which were mixed and expanded, resulting in a population of cells bearing the same dCK variant inserted in different genomic sites (“polyclonal population”). For each HEK-293T clone selected, 4 to 9 independent “polyclonal populations” of Messa10K cells were created and analyzed. (PDF) [file pgen.1002904.s001.pdf]

**Fig. S2**

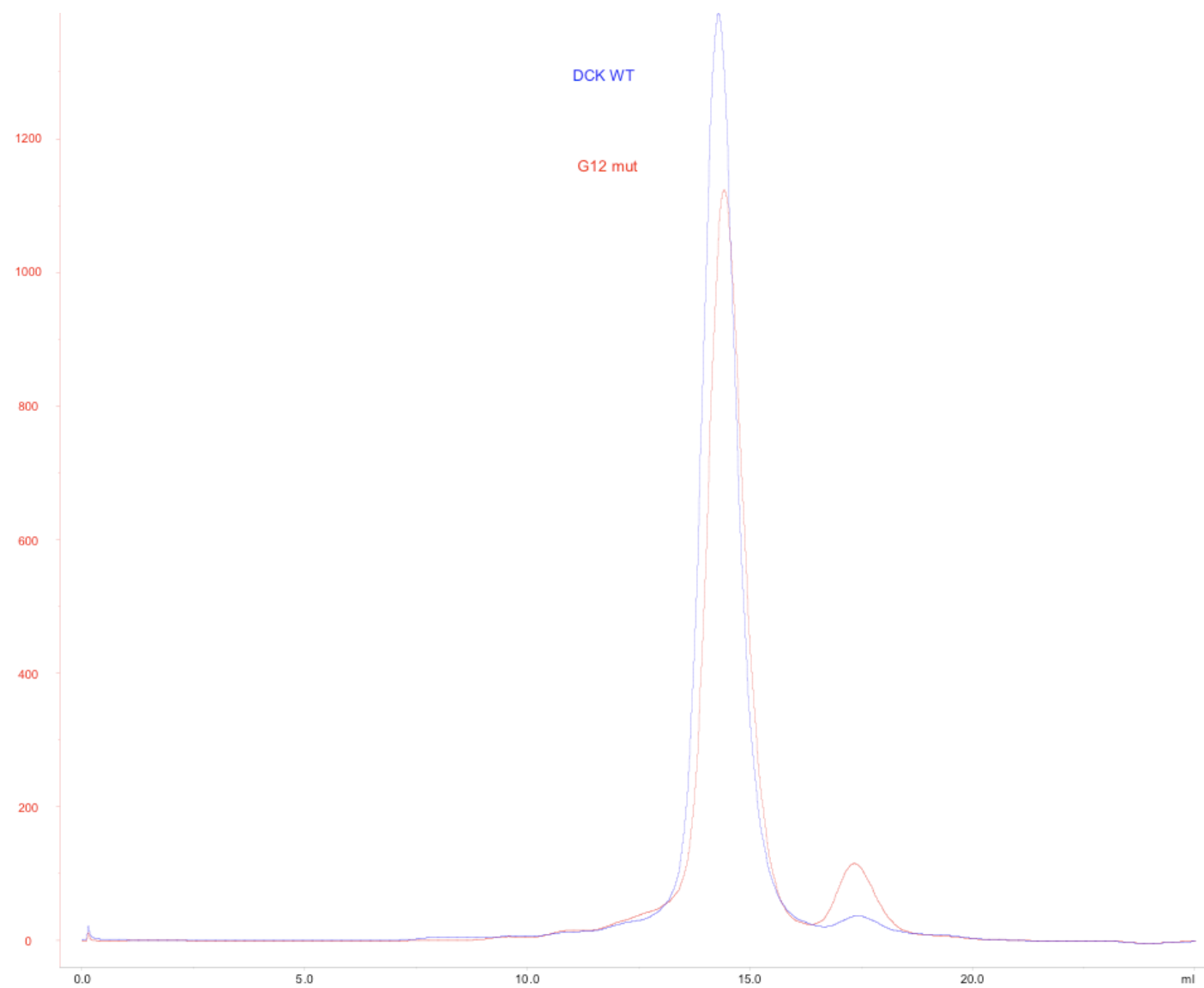

Supplement: Figure S2 — Gel filtration elution profile of wt and G12 dCK proteins. (PDF) [file pgen.1002904.s002.pdf]
